# Macronutrient and Micronutrient Intake in Children with Lung Disease

**DOI:** 10.3390/nu15194142

**Published:** 2023-09-25

**Authors:** Nicole Knebusch, Marwa Mansour, Stephanie Vazquez, Jorge A. Coss-Bu

**Affiliations:** 1 Division of Critical Care Medicine, Department of Pediatrics, Baylor College of Medicine, Houston, TX 77030, USA; nxknebus@texaschildrens.org (N.K.); msmansou@texaschildrens.org (M.M.); stephanie.vazquez@bcm.edu (S.V.); 2 Texas Children’s Hospital, Houston, TX 77030, USA

**Keywords:** children, critically ill, nutrition support, lung disease, metabolism, micronutrients, macronutrients

## Abstract

This review article aims to summarize the literature findings regarding the role of micronutrients in children with lung disease. The nutritional and respiratory statuses of critically ill children are interrelated, and malnutrition is commonly associated with respiratory failure. The most recent nutrition support guidelines for critically ill children have recommended an adequate macronutrient intake in the first week of admission due to its association with good outcomes. In children with lung disease, it is important not to exceed the proportion of carbohydrates in the diet to avoid increased carbon dioxide production and increased work of breathing, which potentially could delay the weaning of the ventilator. Indirect calorimetry can guide the process of estimating adequate caloric intake and adjusting the proportion of carbohydrates in the diet based on the results of the respiratory quotient. Micronutrients, including vitamins, trace elements, and others, have been shown to play a role in the structure and function of the immune system, antioxidant properties, and the production of antimicrobial proteins supporting the defense mechanisms against infections. Sufficient levels of micronutrients and adequate supplementation have been associated with better outcomes in children with lung diseases, including pneumonia, cystic fibrosis, asthma, bronchiolitis, and acute respiratory failure.

## 1. Introduction

The nutritional and respiratory statuses of critically ill patients are interrelated in such a way that they are interdependent while maintaining a balance. Malnutrition is common in pediatric intensive care unit (PICU) patients and is frequently associated with respiratory failure [1,2,3,4,5,6,7,8,9,10,11]. Calorie and protein restriction lead to abnormalities in ventilatory control [12], respiratory muscle function [13], pulmonary structural changes [14], and impaired pulmonary defense mechanisms [15]. In short periods of caloric–protein restriction, metabolic functions of the lung are altered, while extended periods contribute to chronic lung disease, which in turn worsens an already-dismal nutritional condition. Therefore, assessing the nutritional status of patients upon admission to the PICU is imperative, as specified in the latest nutritional support guidelines for critically ill pediatric patients [11]. The nutritional assessment identifies patients at risk, including those with sepsis, acute respiratory failure, and acute kidney injury [5,6,7,16,17].

Various components of the respiratory system are affected by nutrition support, including central stimulation, respiratory muscle function, lung parenchyma, and changes in metabolic demand induced by the consumption of substrates (protein, carbohydrates, and fat). Alterations in respiratory muscle function and structure lead to changes in energy consumption and metabolism [18], so in critically ill patients, the relationship between nutritional status and respiratory failure is of paramount importance to the patient’s health.

It has been well established that micronutrients play an integral and mutually synergistic role in maintaining the structural and functional integrity of both the innate and adaptive immune systems [19,20]. Vitamins A and D are involved in epithelial maturation and production of antimicrobial proteins, respectively. These functions support the gut and lung’s defense mechanisms against infections [19,20,21,22], suggesting that vitamin D deficiency may be associated with a higher risk of respiratory infections [23]. Vitamins C and E support the innate immune system by protecting the epithelial barriers via the induction of collagen synthesis, deactivation of free radicals, and promotion of fibroblast and keratinocyte proliferation and maturation [24,25].

The current review aims to summarize the recommendations for macronutrient intake, the effects of malnutrition on respiratory function, the indications of indirect calorimetry in children admitted to the pediatric intensive care unit, and the results of the supplementation of micronutrients in children with several lung conditions and its effects on lung function and outcomes.

## 2. Methods

A comprehensive literature search was performed on June 18, 2023, for all papers published up to this date, using PubMed, MEDLINE, Embase databases, and Cochrane Library. The terms searched (MeSH heading) included children, critically ill, nutrition support, lung disease, metabolism, macronutrients, and micronutrients. Conference abstracts, case reports, editorials, and non-English language articles were not included unless an abstract was available in the English language. Review papers were searched comprehensively to identify relevant articles. All articles relevant to the objectives of this review were approved by all authors.

## 3. Effects of Malnutrition on the Diaphragm and Respiratory Function

The effects of undernutrition and malnutrition on the structure and function of the respiratory muscles including the diaphragm were published in adults admitted to the intensive care unit receiving mechanical ventilation [26,27,28]. Studies in critically ill adults have described a condition called ventilator-induced diaphragmatic dysfunction with loss of diaphragmatic force-generating capacity and muscle fiber atrophy, myofibril necrosis, and disorganization [29,30,31]. Studies in critically ill children admitted to the pediatric intensive care unit receiving ventilatory support have reported similar findings as the adults’ studies, with diaphragm atrophy and loss of contractility [32,33,34,35,36,37,38].

Malnutrition and inadequate nutrition intake in critically ill children during admission to the hospital are associated with worse clinical outcomes [11,16,39]. Studies in animals exposed to undernutrition have shown atrophy of the diaphragm and intercostals and reduced amount of elastic and collagen fibers in the alveolar septa [40,41,42]. Ultrasonography has been reported as a useful tool in the evaluation of the structure and function of the diaphragm and other skeletal muscles in critically ill children [43,44,45,46,47,48] A report by Koskelo, EK et al. used ultrasonography to measure muscles in the arms and thighs of 16 children with malignancies; the authors concluded that the ultrasound method combined with anthropometry was useful in the assessment of the nutritional status of children [45]. Two reports concluded that the use of ultrasonography in children to evaluate skeletal muscle thickness was not reliable and more studies were needed before muscle ultrasound is routinely used in critically ill children [44,46]. A recent study by Güngör S [49], compared the diaphragm thickness (DT) in children with primary malnutrition and a healthy control group; the authors found that the right and left DT were thinner in the malnourished group vs. the control group and there was a significant weak positive correlation between weight and height z score and right and left DT; r = 0.297, *p* < 0.001; r = 0.301, *p* < 0.001, respectively.

Inadequate nutritional status, increased energy expenditure, airway infection and inflammation, and progression of lung disease are the main characteristics of cystic fibrosis (CF) with malnutrition and chronic lung disease being inextricably interconnected [50,51,52,53,54,55]. An analysis of the Cystic Fibrosis Foundation National CF Patient Registry reported longitudinal relationships between lung function and growth in 968 children over a period of 4 years; the authors concluded that growth and nutritional status are associated with changes in the percent of predicted forced expiratory volume in 1 s (FEV 1%) and that nutritional intervention may slow the decline in lung function in this population [56] A study by Konstan, MW et al. [57] followed a total of 931 children from the ages of 3 to 6 years; the results showed that weight-for-age, height-for-age, and % ideal body weight at 3 years of age were poorly associated with lung disease, but were significantly associated with pulmonary function (forced vital capacity and FEV 1%) at the age of 6 years. The authors concluded that early aggressive intervention aimed at growth and nutrition may affect pulmonary function. Another study by Hart N et al. [58] evaluated diaphragm strength by measuring twitch transdiaphragmatic pressure (Tw Pdi) in 20 patients with CF aged 15.1 ± 2.8 years (SD); the results showed a positive and significant correlation between Tw Pdi and fat-free mass, arm muscle circumference, body mass index, and FEV 1%. These findings indicated that diaphragm strength is preserved in young patients with CF. A study by Hauschild DB et al. [59], evaluated prospectively the association between nutritional status and lung function (FEV 1%) in 38 children (aged 1–15 years, median age of 3.8 years) with CF over a period of 36 months; the results showed that children with nutritional failure at baseline (weight for length and or BMI < 10th) had a relative risk of 5.00 (95% C.I. 1.49–16.76) to have impaired lung function after 36 months. These results indicated that nutritional status was associated with impaired lung function.

The early years in infancy represent a critical period for the development of respiratory diseases later in life [60], and children with malnutrition are more susceptible to repeated and serious episodes of respiratory diseases [61]. Published evidence suggests that children born prematurely have an increased risk of chronic obstructive respiratory diseases in adulthood [62]. Two studies from developing countries reported the association of nutritional status with wheezing and lung function; Hawlader MDH et al. [63], studied 912 children (average age of 4.5 years) in rural Bangladesh and reported that wheezing was significantly associated with stunting and underweight. Ferdous F et al. [64] measured lung function (FEV 1%) in 517 children who had been followed since birth until the age of 9 years; the study found that children who were stunted had lower FEV 1% values compared to children with normal stature. Three large population-based studies evaluated the associations of early childhood growth patterns with lung function and asthma [65,66,67]. The study by den Dekker HT et al. [66] evaluated 24,938 children (age range, 3.9–19.1 years); the results showed that children born with a younger gestational age and children born with a smaller size for gestational age had lower FEV 1% values and had an increased risk of childhood asthma, and greater infant weight gain was associated with higher FEV 1% and risk of asthma. The report by Casas M et al. [65] evaluated 4435 children and performed spirometry at the age of 10 years; the results showed that greater peak weight velocity and body mass index at adiposity peak were associated with lower airway patency in relation to lung volume. A recent study by Voraphani N et al. [67], analyzed data from 652 participants who had at least one set of spirometry data (obtained at ages 22, 26, 32, and 36 years); results showed that maternal nutritional problems during pregnancy, being born small for gestational age, and being underweight in childhood were independent predictors of spirometric restriction in adult life.

## 4. Caloric and Protein Needs of the Patient with Pulmonary Disease

Based on the nutritional support guidelines of ASPEN (American Society for Parenteral and Enteral Nutrition) (American Society for Parenteral and Enteral Nutrition) and SCCM (Society of Critical Care Medicine), indirect calorimetry can be used to determine the energy requirements of pediatric patients with acute respiratory failure [11]. When indirect calorimetry cannot be used, the use of formulas to estimate basal metabolism is recommended to determine the caloric requirement for each patient [68]. In pediatric patients, the Schofield formula should be used. In addition, it is important to emphasize the inappropriate use of correction factors during the acute phase of the disease as well as the possibility of excessive caloric intake [69,70,71,72]. Following the acute phase of the disease, the caloric intake should be estimated based on the child’s nutritional status and age. Infants and young children will need more calories to maintain adequate growth and development. 

During the acute phase, protein intake is essential due to the increased degradation of proteins [1,2,70,73,74,75,76,77,78]. Age plays a crucial factor since infants and young children require a higher protein intake per kilogram of body weight. According to ASPEN/SCCM, a minimum protein intake of 1.5 g/kg/day is recommended, and this amount should be adjusted based on the levels of biomarkers, such as prealbumin and *C*-reactive protein [1,11].

The goals of nutritional support in the patient with lung disease include (1) adequate caloric intake, (2) adequate protein intake to prevent muscle loss, (3) correction of the cause of respiratory failure, (4) avoidance of excess carbon dioxide production, and (5) reversal of the nutritional-related sequelae of lung disease, which includes optimization of exercise tolerance and normalization of growth, which are particularly relevant in infants and young children [79].

## 5. Use of Indirect Calorimetry for Optimization of Nutritional Support

Nutrient administration has effects at the level of the respiratory system through physiological and pharmacological processes. An increase in dietary carbohydrate intake increases ventilatory demand secondary to an increase in carbon dioxide production (VCO_2_) [80,81,82]. Lipids can have an impact on the vascular tone and inflammatory response of the pulmonary vascular system by serving as precursors of eicosanoid synthesis [83]. Amino acids can increase oxygen consumption (VO_2_) and stimulate ventilation through modifications of the respiratory drive [84].

Indirect calorimetry (IC), which uses the gas exchange method, determines the caloric equivalent of VO_2_ and VCO_2_. For the measurement of energy expenditure in hospitalized patients, this method is considered the gold standard [2,3,73,85,86,87,88,89,90]. IC allows for the calculation of the relative oxidation of the different substrates (carbohydrate, protein, and fat) by obtaining the value of total nitrogen in urine and the use of the respiratory quotient (RQ). Depending on the type of oxidation taking place, the RQ value can range from 0.71 for fatty acid oxidation to 1.0 for carbohydrate oxidation. A value greater than 1.0 indicates a process of lipogenesis, while a value of 0.81 indicates protein oxidation. 

Several studies have reported the importance of indirect calorimetry and RQ in caloric adjustment and diet composition in relation to the amount of carbohydrate and fat [2,91,92,93,94,95,96]. The results suggest that the use of IC is necessary for individual adjustment of nutritional support of critically ill patients, particularly those suffering from pulmonary diseases. An excess of carbohydrates will increase breathing effort due to increased carbon dioxide production, resulting in difficulty weaning patients off ventilators.

There have been mixed results when RQ has been used as an indicator of excessive or insufficient caloric intake in critically ill pediatric patients [91,92,93,94,96]. According to Mehta, NM et al. [96], RQ results were obtained for 14 pediatric patients admitted to the PICU. The RQ in hypermetabolic patients [*n* = 7, 50 ± 64 (SD) kg] was 0.85 ± 0.03 with a caloric intake of 1464 ± 1008 kcal/day, while hypometabolic patients (*n* = 7, 45 ± 59 kg), had an RQ value of 0.94 ± 0.06 with a caloric intake of 935 ± 559 kcal/day. The authors highlighted the importance of using IC for individualized guidance on nutritional support. Three studies reported sensitivity and specificity values of RQ in pediatric patients as an indicator of excess or lack of caloric intake. The study by Hulst, JM et al. [92] of 95 patients admitted to PICU showed sensitivity and specificity for an RQ value < 0.85 (lack of caloric intake) of 63% and 89%, respectively, while sensitivity and specificity for an RQ value > 1.0 (excess caloric intake) were 21% and 97%, respectively. The report by Dokken M. et al. [91] of 30 mechanically ventilated patients admitted to PICU, reported for an RQ value <0.85 a sensitivity and specificity of 27% and 87%, respectively, and for RQ value > 1.0 a sensitivity and specificity of 21% and 98%, respectively. Liusuwan RA et al. [94], in 74 pediatric patients with burns of >20% body surface area reported: for an RQ value < 0.85, a sensitivity and specificity of 40% and 77%, respectively; and for an RQ value > 1.0 a sensitivity and specificity of 23% and 85%, respectively. The report by Kerklaan D et al. [93] of 78 mechanically ventilated pediatric patients concluded that the identification of patients with an excess or lack of caloric intake using IC and RQ values depended on the definition used to categorize the patients. Thus, it can be concluded that the RQ value identified as an excess or inadequate caloric intake in critically ill pediatric patients has low sensitivity and adequate specificity.

## 6. Micronutrients and Their Associations with Lung Function and Disease

Vitamins, trace elements, and other micronutrients have different functions in the body homeostasis, and participate in the production of enzymes and in the balance of the immune system (Table 1). Vitamin D modulates the immune system, including the innate and adaptive immune systems, and improves pulmonary function by stimulating antimicrobial peptide production [97,98,99]. Whereas vitamin A is involved in cell integrity, growth, and immune function [100], vitamin E serves as an antioxidant, and vitamin K is needed for blood clotting and metabolic functions [101,102]. The Recommended Dietary Allowances and Adequate Intakes for Vitamins and other micronutrients are listed on Table 2.

### 6.1. Pneumonia

Pneumonia is the leading cause of pediatric hospitalization and death in children under 5 years of age worldwide. Malnutrition is a major risk factor for the development and severity of pneumonia. Though we have made major advances in reducing mortality, malnutrition remains a poor prognosis marker with over half of in-patient pneumonia deaths caused by malnutrition. Not only is optimizing macronutrients in these patients vital for recovery, but so is taking a closer look at micronutrients and the role supplementation can play in prevention and treatment [126]. 

Early research found vitamin C deficiency to be in parallel with severe respiratory infection, specifically pneumonia. Scurvy has been associated with pneumonia, and the last one has been the reported cause of death. This led to further investigation of the possible benefits of vitamin C supplementation for the prevention and/or treatment of pneumonia. Even with normal levels of vitamin C, levels decrease significantly during respiratory infections as its antioxidant properties are needed to combat oxidative stress. Multiple studies have shown a strong correlation between vitamin C deficiency and lower respiratory infections [127]. 

Multiple studies have shown an association with the use of vitamin C supplementation to decrease the severity of upper respiratory infections, but no significant differences have been seen to prevent lower respiratory infections such as pneumonia [128]. A meta-analysis performed by Hemila H et al. examined the effect of vitamin C supplementation in preventing and treating pneumonia. Three studies showed more than an 80% decrease in the incidence of pneumonia and a decrease in mortality in patients receiving vitamin C, especially with those with low plasma vitamin C levels [129]. Most recently, Padhani ZA et al. analyzed further, looking into five RCT studies, which included 2655 participants, which included pediatric population in most of the studies reviewed. Two studies focused on pneumonia prevention with vitamin C supplementation doses of 1 g daily for 14 weeks, 2 g daily for 8 weeks, and 2 g daily for 14 weeks. Three studies provided vitamin C supplements as an adjunct therapy for pneumonia treatment, using a dose of 125 mg daily and 200 mg daily until the symptoms resolved or discharged. Though some studies did show that the vitamin C group had shorter hospital durations, evidence for the use of vitamin C supplementation for the prevention or treatment of pneumonia was very uncertain. Further studies are needed to assess the effectiveness of the possible use of vitamin C in treating and preventing pneumonia [130]. 

There are a significant number of studies describing the association between low vitamin D levels and increased risk of lower respiratory infections in the pediatric population. Multiple RCTs across the world have been performed comparing vitamin D levels of children with lower respiratory tract infections including pneumonia vs. healthy control group. Insufficient or vitamin D-deficient plasma levels were associated with increased complications and severity of disease such as escalation in respiratory support, requiring mechanical ventilation support, admission to the pediatric intensive care unit, and prolonged length of hospital stay [131,132,133,134,135].

A recent analysis by Das RR et al. looked at vitamin D as an adjunct to antibiotics for the treatment of acute childhood pneumonia; seven RCTs, which included 1601 hospitalized children (ages 1 month to five years old) with pneumonia, compared vitamin D supplementation vs. the placebo group. The dosage of vitamin D varied, with five trials using a single dose of 100,000 to 300,000 IU within 24 h of admission, another trial using a daily dose (1000 IU for ages <1 year and 2000 IU for ages >1 year) for five days, and the last trial using different doses on day 1 (20,000–100,000 IU depending on age) followed by 10,000 IU daily for four more days. No significant difference was observed in the duration of illness, duration of hospitalization, or mortality. The evidence was low-to-moderate certainty for all outcomes [136]. 

Though further studies need to be performed to demonstrate any benefit of using vitamin D for treating pneumonia. Prevention of lower respiratory tract infections with regular vitamin D supplementation has been shown to make a difference. RCTs in Japan found that a daily dose of 1200 IU of vitamin D for school-age children during winter months decreased the incidence of influenza [137]. Other studies have investigated children receiving regular milk fortified with vitamin D and were observed to have decreased incidences of respiratory tract infections [138]. Also, infants who received the recommended vitamin D supplement 400–600 IU up to 6 months of age were less likely to have respiratory tract infection compared to infants who did not receive vitamin D supplement [139]. It is evident there is a correlation between lower vitamin D levels and the risk of a respiratory infection such as pneumonia, though further studies are needed to determine the appropriate dose to achieve the protective immune effect that has been observed. 

A large meta-analysis that included over a million non-hospitalized children aged 6 months to 5 years old reported that vitamin A supplementation was associated with a decrease in morbidity and mortality in children. Regarding respiratory diseases such as pneumonia, there was no significant correlation or benefit observed. Due to the strong evidence of the effect on the reduction in mortality, all children at risk of vitamin A deficiency are recommended to take a supplement as dictated by the RDA [140].

A prospective cohort study in 2018 obtained serum zinc levels in pediatric patients admitted to the pediatric intensive care unit with severe pneumonia and pediatric patients with pneumonia admitted to the non-intensive care unit. The study found there was a significant difference between the two groups; with decreased serum zinc levels observed in the patients admitted to PICU compared to those admitted to the pediatric floor. Of note, zinc levels were significantly lower in critically ill children whose pneumonia was complicated by sepsis, ventilation support, and/or death [141]. An RCT was carried out with 103 hospitalized children (1 month to 5 years old) with pneumonia, where zinc supplementation (10 mg/day if <1 year and 20 mg/day if >1 year) vs. placebo was given. Children who received zinc supplements had faster clinical improvement along with improved oxygen saturation and respiratory rate [142]. Another RCT with 100 hospitalized children (28 days to 5 years old) with pneumonia where zinc supplementation (20 mg/day) vs. placebo was given. This study found no differences in symptoms, but the zinc supplementation group had a significantly shorter hospital admission [143]. Despite some studies showing potential in the use of zinc to treat pneumonia, the evidence is inconclusive and limited.

In contrast to the evidence of using zinc for the treatment of pneumonia, various studies have shown the benefit of zinc supplementation for the prevention of lower respiratory infections such as pneumonia. A review of six RCT studies that included 5193 children (aged 2 months to 59 months) evaluated zinc supplementation at doses recommended by the RDA to prevent pneumonia; the results showed a decreased incidence (13%) and prevalence (41%) of pneumonia in those who received zinc supplementation [144]. 

### 6.2. Cystic Fibrosis 

A child with cystic fibrosis (CF) is likely to have insufficient or deficient levels of fat-soluble vitamins because of the malabsorption of fat-soluble vitamins caused by exocrine pancreatic insufficiency, poor nutritional intake, changes in metabolism, and other factors [99,145,146]. These vitamins are routinely supplemented to CF patients to avoid deficiencies. Normal laboratory values for several micronutrients are presented in Table 3. Many patients with CF have low levels of vitamin D, with one study reporting that 40% of children with CF had deficient levels of vitamin D [146]. A study evaluating fat-soluble levels of 556 children less than 18 years of age reported that 15% presented with vitamin A deficiency and 20% vitamin E deficiency, and while only 14 samples were taken for vitamin K, 29% presented vitamin K deficiency [147].

The presence of inadequate and deficient levels of vitamin D has been associated with increased pulmonary exacerbations in children with cystic fibrosis. A study by McCauley LA et al. found that adolescents aged 15–18 years with vitamin D deficiency had a higher rate of exacerbations than those with insufficient or sufficient levels of vitamin D, 13 per 10 patient-years (95% CI, 6–31; *p* < 0.05) vs. 4.3 per 10 patient-years (95% CI, 2–8; *p* < 0.05), respectively [149]. A report by Aziz D et al. of 69 children with CF aged 3 to 18 years found that children with vitamin D deficiency had significantly more pulmonary exacerbations per year than patients with insufficient or sufficient levels (*n* = 28, 3.71 ± 0.96; *n* = 22, 3.18 ± 1.09; and *n* = 19, 2.26 ± 0.93; respectively; *p* < 0.001 [145]. In another study, 148 children from 10 months to 12 years of age were evaluated for inflammation and bacterial colonization. Results showed that infection with *p*. Aeruginosa was more common in children with deficient or insufficient levels of vitamin D compared to children with sufficient levels of vitamin D (18 of 63 vs. 11 of 85, respectively; *p* = 0.018). This study also demonstrated that median serum 25-hydroxyvitamin D (25-OHD) concentrations in patients colonized with *p*. Aeruginosa were significantly lower comparted to patients without the infection (27.7 ng/mL; interquartile range [IQR], 25.3–33.8; vs. 32.9 ng/mL; IQR, 26.5–39.3; *p* = 0.021) [98]. While it is evident that children with adequate levels of vitamin D have benefits regarding colonization and exacerbations, it is necessary to conduct larger sample size studies to determine whether current recommendations are appropriate.

Regarding micronutrient levels and their association with pulmonary function, several retrospective studies have demonstrated a relationship between vitamin D status and predicted forced expiratory volume in 1 s (FEV1%). Based on the results of the study by Timmers N et al., a 20 nmol/L increase in serum levels of 25-OHD in children with CF increased FEV1% prediction by 1–12% (95% CI 0.2, 2.04) [146]. Another study by McCauley L et al. reported that an increase of 10 mg/L of 25-OHD in 16-year-old children with CF was associated with a 5.5% increase in predicted FEV1 (95% CI; 0.5–10.5%; *p* = 0.036), even though these results were not statistically significant in younger children [149]. A pulmonary function test was performed in another study by Woestenenk JW et al., evaluating vitamin E levels in children with CF and predicted FEV1%, and no significant association was found [102]. As for vitamin A, a study performed by Loukou L et al. on 231 pediatric patients with CF showed that serum retinol levels were positively associated with predicted FEV1%, showing that with an increase of 1 ug/dL of serum retinol level, there was an increase of 0.39 in predicted FEV1% [105]. Having sufficient serum levels of fat-soluble vitamins is extremely important in this population to have an optimal pulmonary function and prevention of exacerbations, but still, no evidence has been found of the direct effects of supplementation during pulmonary exacerbation. 

A stable concentration of various micronutrients is needed to maintain homeostasis. Zinc is an important micronutrient for enzymes, cellular transporters, growth, metabolism, and development [150]. In a study by AbdulWahab, A. et al., pulmonary function as measured by FEV1 in children with CF was lower, but not statistically significant, in pediatric patients with zinc deficiency vs. patients with normal levels; 66.5 ± 29.6% vs. 81.4 ± 20.4%, *p* = 0.133, respectively [151]. A double-blind placebo-controlled study by Abdulhamid I et al. evaluated supplementation with 30 mg/d of zinc vs. placebo in 26 children with CF. The results showed that children with baseline zinc deficiency before supplementation had a significant decrease in the number of days of oral antibiotics use per year, compared to the placebo group (*p* < 0.05) [152]. Although a similar study was performed by Sharma G et al., no effect in days of antibiotic use in children who received 30 mg/d of zinc supplementation was reported [153].

Regarding omega 3 supplementation in CF, one metanalysis of five studies reported inconclusive results regarding supplementation, as some studies reported no difference in lung function or antibiotic use and one study reported a decrease in pulmonary exacerbations and antibiotic use when comparing supplementation vs. placebo [154]. Antioxidant micronutrients can help maintain balance in oxidative stress, a metanalysis of 19 studies analyzed the effects of antioxidant supplementation in CF patients; only 1 study reported that after 2 months of supplementation with vitamin E, C, A, beta carotene, and selenium, there was an increase in predicted FEV1% compared to the control group, MD −4.30% (95% CI −5.64 to −2.96) [155]. These results show that supplementation with antioxidants might be beneficial in the short term, but there is a lack of studies evaluating the benefits and outcomes of long-term supplementation of zinc, omega 3, and other antioxidants in children with cystic fibrosis. More studies are needed to establish a recommendation about additional supplementation during pulmonary exacerbations.

### 6.3. Asthma

A deficiency of vitamins can adversely affect the immune system, thus leading to a worsening of pulmonary functions and asthma management [156]. Numerous studies have demonstrated an association between vitamin deficiencies and asthma severity [157,158]. The results of a meta-analysis by Jat, K.R et al. showed that children with asthma have an increased risk of vitamin D deficiency by 3.4 times (OR 3.41, 95% CI 2.04–5.69, *p* < 0.0001) [158]. A deficiency of vitamin E has not been linked to asthma prevalence, but a low level of vitamin C serum has been linked to severe asthma (*p* < 0.001) [157,159]. According to the meta-analysis cited above, there is no association between vitamin D levels and pulmonary function as measured by FEV1 or FEV1/FVC [19]. However, there is a weak positive correlation between vitamin C levels and FEV1/FVC ratio (r = 0.35; *p* = 0.002) [157]. A case-control study by Anand V et al. revealed a higher risk of severe asthma among children lacking adequate vitamin D levels (OR 2.81, 95% CI 1.36–5.82) [160]; another observational study of 114 pediatric patients demonstrated a higher prevalence of vitamin D insufficiency in children with severe asthma compared to children with moderate asthma (*p* = 0.013) [161]. The results of this study are like those reported by Wu et al., who conducted a systematic review in children, and no associations were found between vitamin E supplementation and asthma [159]. Although these associations have been observed, no difference has been found between vitamin D supplementation and asthma control in terms of the number of exacerbations, number of exacerbation days, emergency visits, hospital admissions, or the use of medication when compared to placebo [156,162]. 

Regarding trace elements and asthma control, Kuti BP et al. reported in a cross-sectional study of 160 children that subjects with asthma have lower levels of zinc and selenium than control children (*p* = 0.008 and *p* = 0.033, respectively); they also found an association between low serum selenium levels and moderate-to-severe asthma (*p* < 0.001), but no such association was observed with zinc and asthma severity [163]. These results can be compared to those shown by Rajkumar et al., which did not find an association between severity of asthma and zinc levels below 60 µg/dL. However, this study found a significant difference in zinc levels between asthma patients with controlled and uncontrolled asthma (*p* = 0.006), indicating that children with higher zinc levels had better symptom control [164]. In addition, zinc plasma concentrations have a positive association with FEV1 and FEV1/FVC ratios as reported by Siripornpanich S. et al. (r = 0.27, *p* = 0.019, and r = 0.65, *p* = 0.001, respectively) [157]. An appropriate level of zinc is beneficial in asthmatic children; however, more studies are needed to determine optimal zinc levels and the optimal supplementation dose for children with asthma.

The benefits of antioxidants and its anti-inflammatory properties were evaluated by Sdona E. et al. in a population-based birth cohort BAMSE study; a total of 2307 Swedish children with asthma were evaluated, and it was found that higher consumption of antioxidants as measured by the total antioxidant capacity at 8 years of age was associated with a higher FEV1 at age 16 (0.46 SD, 95% CI 0.11–0.8). Also, there was a significant difference between having a higher total antioxidant capacity at age 8 and having a lower likelihood of low lung function at age 16 (OR 0.28, 95% CI 0.12–0.65; *p* = 0.008) [165]. A cross-sectional study by Farjadian S. et al. of 39 children with asthma showed improvement in FEV1/FVC ratios after consuming 180 mg of eicosapentaenoic acid (EPA) and 120 mg of docosahexaenoic acid (DHA) daily for three months (*p* = 0.044) [166]. The presence of antioxidants and omega 3 fatty acids in the body has been shown to provide long-term health benefits for children who suffer from asthma. To determine the appropriate dosing and timing, more studies are needed.

### 6.4. Bronchiolitis and Acute Lower Respiratory Tract Infection

Acute lower respiratory tract infection (ALRTI) has been reported worldwide as a leading cause of morbidity and mortality in children less than 5 years of age, and approximately 1 in 5 of those deaths in those children are ascribed to ALRTI [167,168]. 

A study by Alakas Y et al. evaluated the relationship between Vit D (25-OHD) levels and disease severity in 182 children with diagnosis of bronchiolitis; almost half (47.8%) of the children were Vit D-insufficient (12–20 ng/mL) or -deficient (<12 ng/mL) and children with low Vit D levels had a higher percentage of patients with severe disease or admission to the intensive care unit [169]. A case-control study by Roth DE et al. reported Vit D (25-OHD) status in 129 children aged 1–25 months admitted to the hospital for uncomplicated ALTRI (primarily bronchiolitis); the study reported similar levels of 25-OHD among cases and controls (77.0 vs. 77.2 nmol/L, *p* = 0.960); the authors concluded no association between levels of Vit D and risk of hospitalization [170]. In a secondary analysis by Toivonen L et al. [171] of a database comprising 1016 children aged less than 1 y and hospitalized with bronchiolitis, levels of 25-OHD were dichotomized into lower (<26.5 ng/mL) and higher (>26.5 ng/mL) groups and correlated with nasopharyngeal microbiota profiles. In infants with lower 25-OHD levels, the Haemophilus-dominant microbiota profile was associated with a significantly higher risk of intensive care use (OR: 3.08, 95%CI: 1.31–7.25, *p* = 0.01) vs. the Moraxella-dominant profile. The authors concluded that a significant interaction was present between serum total 25-OHD levels and nasopharyngeal microbiota on bronchiolitis severity. A report by Doumant G et al. evaluated the association between levels of 25-OHD and lung function in 363 children at age 3 and 6 y with a history of bronchiolitis. At age 6, comparing the highest quintile (Q5) of serum 25-OHD (median 37 ng/mL) vs. the lowest quintile (Q1; median 18 ng/mL), FEVI% was 6% lower (*p* = 0.03) in Q1 and forced vital capacity was 7% lower in (*p* = 0.03) in Q1; the authors concluded that higher vitamin D status correlates with better lung function [172]. A study by Khoshnevisasl *p* et al. randomized 94 infants aged 2 to 23 months into 3 groups: hypertonic saline (control), vitamin D (100 units/kg/day) or zinc (20 mg/day). On the third day of hospitalization, the respiratory rate among the three groups was not statistically significant, nor was the duration of hospitalization statistically different [173]. A meta-analysis by Das RR et al. evaluated the short therapeutic role of zinc in 1066 hospitalized children in developing countries; the results showed no significant difference between the zinc vs. the placebo group regarding the time to resolution of severe illness (standardized mean difference (SMD) −0.15 (95%CI −0.5–0.2; *p* = 0.4)) and duration of hospitalization (SMD −0.29 (95%CI −0.68–−0.09; *p* = 0.13). These results do not support the efficacy of zinc supplementation for severe ALRTI [174]. A recent RCT by Ahadi A et al. included 100 children aged 2 to 32 months with diagnosis of bronchiolitis; 50 patients received zinc supplementation on admission and 50 patients received placebo. At 72 hrs after admission, the difference between the two groups was statistically significant in terms of the number of people with wheezing (zinc vs. placebo, 16% vs. 36% (*p* = 0.023)) and rhinorrhea (0% vs. 12% (*p* = 0.027)); and the hospital length of stay was shorter in the treatment group vs. the placebo: 4.14 ± 1.21 (SD) vs. 4.64 ± 1.20 days, *p* = 0.016 [175]. A case control study by Gurkan F et al. evaluated the association of selenium levels in 59 children: 34 with bronchiolitis and 25 controls. The selenium levels were measured during the acute period of bronchiolitis and 2 months after the discharge from the hospital. The selenium levels at time of hospitalization, post-discharge, and of the control group were 32 ± 29 µg/L, 68 ± 26 µg/L, and 145 µg/L, respectively, *p*< 0.001 [176].

### 6.5. Acute Lung Injury and Pediatric Acute Respiratory Distress Syndrome (ARDS)

The systemic inflammatory response (SIRS) triggers oxidative stress, as seen in acute respiratory distress syndrome, both in adults and children [177,178]. A report by Valla FV et al. reported micronutrient status in 99 critically ill children with organ dysfunction, including respiratory failure, and compared it with 102 healthy controls; the results showed a significant trend (*p* < 0.02) toward a plasma level decrease in six micronutrients (selenium, zinc, cooper, vitamin E, vitamin C, and β-carotene), while oxidative stress increased. The authors concluded that identification of critically ill children with severe oxidative stress will help in identifying the patients that can benefit the most from micronutrient supplementation [179].

Vitamin C is a micronutrient with many physiologic properties, including a cofactor for enzymes involved in protein and hormone synthesis, metabolic pathways for energy generation, and regulation of gene transcription [180,181]. Thiamine is involved in several stages of intermediate metabolism aimed at the production of energy, and acts as a cofactor in oxidative decarboxylation in three mitochondrial complexes [182]. Two studies have reported levels of vitamin C and thiamine in critically ill children; the report from de Lima LFP et al. reported in 202 critically ill children a prevalence of low thiamine of 28% [182], while Fathi A et al. reported in 60 critically ill children a vitamin C deficiency prevalence of 18% [183]. The combination of hydrocortisone, intravenous ascorbic acid (vitamin C), and thiamine has been proposed as an adjunctive therapy, primarily targeting the oxidative stress [184]. A retrospective propensity score-matched analysis by Wald EL et al. of 43 children with septic shock who received HAT therapy (hydrocortisone, ascorbic acid, and thiamine) compared to 181 children that received only hydrocortisone and 333 controls showed a lower 30-day mortality of 9% for the HAT group vs. controls (28%) and the hydrocortisone group (30%). The authors concluded that larger, multicenter RCTs are needed in children to confirm their results [185].

Low levels of vitamin D have been identified as a risk factor for cardiovascular disease, muscle weakness, impaired metabolism, and compromised lung function [186]. The prevalence of hypovitaminosis D in critically ill children has been reported from 34.5% to 69% [186,187,188]; two recent systematic reviews showed that low levels of vitamin D in children admitted to the pediatric intensive care unit were associated with illness severity and the need for therapeutic interventions and mortality [189,190]. A systematic literature review by Wang et al. showed that vitamin D deficiency could be associated with longer duration of acute respiratory infections in young adults [191]. A single-center retrospective observational study showed that vitamin D deficiency was prevalent in adults with severe COVID-19-induced ARDS. In that cohort, vitamin D deficiency was associated with increased ICU length of stay and mechanical ventilation without impacting mortality [192]. When the COVID-19 pandemic started, many research endeavors tried to explore the pathophysiology of COVID-19-induced ARDS. Evolving evidence points to COVID-19-induced mitochondrial dysfunction and excessive levels of reactive oxygen species (ROS) leading to a vicious cycle of immune dysregulation, inflammation, and lung injury [193,194]. Zinc, selenium, vitamin E, and other essential micronutrients are well known to have antioxidative effects and play an integral role as co-enzymes in the immune response cytokine cascades and lymphocyte maturation [113,195,196]. 

Zinc is known to modulate the proinflammatory responses affecting natural killer cell activity and cytokine levels for IL-1b, IL-6, and TNF-α [197]. The literature further shows that zinc deficiency is associated with decreased antibody production impairing immune responses and is associated with a higher risk of respiratory viral infections in the zinc-deficient population and elderly individuals [197,198]. Research has further shown that zinc does not only affect immune responses but also affects viral replication. Evidence shows that increasing the intracellular zinc level can disrupt Coronavirus species (CoV) replication, possibly via the inhibition of RNA polymerase activity [199,200,201]. These observations suggest that zinc supplementation may decrease the severity and duration of respiratory symptoms, enhance immune cell maturation and phagocytosis, and improve the response to immunotherapy in various viral infections [197,202].

Research has shown that selenium deficiency is observed in critically ill patients with multiorgan failure [203,204]. Selenium contributes to immune modulation as it enhances the transformation of M1 macrophages (proinflammatory) to M2 (anti-inflammatory) macrophages, enhances IL-2 production, and promotes lymphocyte proliferation [205,206]. Selenium deficiency is associated with decreased production of free radicals and impaired functions of immune cells including neutrophils, T lymphocytes, and natural killer (NK) cells [202]. Selenium is incorporated into antioxidant enzymes and selenoproteins, like glutathione peroxidases and thioredoxin reductases, which play integral roles in reducing viral replication [207].

A retrospective chart review in adults by Notz et al. showed substantially low zinc and selenium levels in patients with severe COVID-19-induced ARDS. In that cohort, the researchers observed restoration of NK and CD8+ T cell subsets with trace element supplementation and increasing levels of Se and Zinc. In addition, improved selenium levels were associated with improved PaO_2_/FIO_2_ ratios, alluding to the potential effect of selenium and zinc levels on the immune response in critically ill patients with severe COVID-19 ARDS [208].

It is well established that vitamin A plays an instrumental role in mucosal immunity. The beneficial effects of vitamin A on the morbidity and mortality of some viral infections, such as measles and HIV, could be due to increased antibody production and lymphocyte proliferation as well as enhanced T-cell lymphopoiesis. [209]. A multicenter prospective study by Tepasse et al. showed that low vitamin A plasma levels correlated with increased levels of inflammatory markers (*C*-Reactive Protein and ferritin) and low lymphocyte count, indicating severe SARS-CoV-2 infection. Patients with low vitamin A levels had a higher likelihood of developing severe ARDS and higher mortality. Hospitalized patients with critical illness showed significantly lower vitamin A levels than those who were moderately ill. The investigators concluded that low vitamin A plasma levels in COVID-19 patients are significantly associated with ARDS and mortality [210]. Table 4 summarizes the important clinical findings of micronutrients in children with several lung conditions.

## 7. Conclusions

In summary, a critically ill pediatric patient’s nutritional and respiratory status are closely related, and changes in one significantly impact the other. Malnutrition and the presence of pulmonary disease have been demonstrated to cause structural and physiologic changes in the respiratory system in clinical trials and animal models. An assessment of nutritional status is imperative upon admission to the PICU, as is the identification and assessment of acute or chronic lung disease. Early nutritional support is of the utmost importance for all patients admitted to the PICU, as well as the use of nutritional support protocols and algorithms. In patients with pulmonary disease, it is important to adapt the macronutrient distribution with a reduction in carbohydrate intake, an age-appropriate amount of protein, and a slight increase in fat intake. This will help prevent an increase in carbon dioxide production and decrease the work of breathing, shortening the time of mechanical ventilation and hospital stay. Micronutrient supplementation including vitamins, trace elements, and other macronutrients has been shown to improve the balance of the immune system, improvement of respiratory function by stimulating antimicrobial peptide production, increasing antioxidant properties, etc. Results of the published literature have shown an association between decreased micronutrient levels and lung disease, and have also shown positive clinical effects of the supplementation of vitamin A and vitamin C in children with pneumonia, improved lung function in children with cystic fibrosis supplemented with vitamin D and a mixture of antioxidants, improved lung function in children with bronchiolitis who had a higher vitamin D status, and a survival benefit in critically ill children supplemented with HAT therapy. These micronutrients can be administered either by an intravenous or enteral route, and appropriate dosage should be administered in the presence of renal or liver dysfunction. Large, randomized control trials are needed in children with lung disease to determine the clinical benefits of micronutrient supplementation.

## Figures and Tables

**Table 1:** Role of micronutrients in immune and pulmonary functions.

Micronutrient	Protective Role	Immune Functions	Lung-Related Functions
Vitamin D [103,104]	Anti-inflammatory Antimicrobial	Downregulates proinflammatory cytokines and increases TH2 cytokines. Contributes to the maintenance of Treg cells.	Increases expression of antimicrobial cathelicidin and airway epithelium response against viruses.
Vitamin A [105,106]	Anti-inflammatory Antioxidant	Maintains cell proliferation, differentiation, and integrity. Induces Treg cells to differentiate immune tolerance.	Maintains respiratory tract epithelial cells. Deficiency is associated with respiratory disease.
Vitamin E [107]	Anti-inflammatory Antioxidant	Deficiency is associated with an increase in inflammatory cytokines.	Constituent of lung surfactant Deficiency has been associated with a pro-apoptotic state of type II lung cells.
Vitamin C [108,109]	Antioxidant	Cofactor for enzymes is present in phagocytes and lymphocytes. Protects cells against oxidative stress.	Decreases eosinophilic infiltration in respiratory tract; decreases inflammation, mucus hypersecretion, and bronchoconstriction.
Selenium [110,111]	Anti-inflammatory Antioxidant	Participates in the synthesis of antioxidants like glutathione peroxidase, protects against apoptosis, increases proliferation and maturation of T cells and natural killer cells.	Cofactor of glutathione peroxidase and able to scavenge reactive oxygen species, which plays a role in the development of asthma through damaging normal tissue of lung
Zinc [112,113]	Anti-inflammatory Antioxidant	Attenuates proinflammatory response, acts as an antioxidant intracellularly, and enhances the function of activated b cells.	Low levels of zinc promote an apoptotic state of respiratory epithelium.
Omega 3 [114]	Anti-inflammatory	Converted to resolvins, protectins, and maresins. Decreases inflammation by decreasing neutrophil migration and decreasing proinflammatory cytokines and chemokines.	Protects against apoptotic states and increases bacterial clearance.
Folate [115,116]	Modulator	Deficiency is associated with decreased CD8+ T cells and increased susceptibility to infections.	May decrease allergic airway inflammation. Decreases reactive oxygen species and Th2 immune response in asthma.
Cobalamin Vitamin B12 [117,118]	Modulator	Involved in synthesis of nucleic acids and synthesis of proteins such as antibodies and immunoglobulins. Deficiency is associated with a decreased number of CD4+ cells.	Suppresses viral replication in host cells.
Pyridoxine Vitamin B6 [118,119]	Modulator	Deficiency is associated with a decrease in proliferation of lymphocytes and reduced IL-2 production.	Decreases inflammation and lipid peroxidation. Alters caspase activation and AMPK phosphorylation which impacts lung inflammation and function.
Thiamine Vitamin B1 [120,121]	Anti-inflammatory	Participates in the production of integrins which affects the immune system’s reactivity. Regulates expression of inflammatory agents.	Improves VO_2_ consumption in critically ill patients.
Copper [122,123]	Antioxidant	Cofactor for enzymes that participate in redox reactions and production of superoxide anions. Deficiency is associated with a decrease in neutrophils and impaired function of B and T-cells.	Cofactor of LOX lysyl oxidase, which maintains and matures elastin fibers collagen production necessary for lung structure and distension.
Riboflavin [124]	Antioxidant	Cofactor for enzymes that participate and regulate oxidative stress. Participates in the activation and modulation of macrophages and neutrophils.	Cofactor of FAD-dependent enzymes which protects lungs from oxidant-mediated injury and inflammatory injury.

**Table 2:** Dietary reference intakes (DRIs): recommended dietary allowances and adequate intakes for vitamins and elements for infants, children, and adolescents.

	Infants 0–6 mos	Infants 7–12 mos	Children 1–3 y	Children 4–8 y	Females 9–13 y	Females 14–18 y	Males 9–13 y	Males 14–18 y
Vitamin A (mcg/d) ^a^	400 *	500 *	300	400	600	700	600	900
Vitamin C (mg/d)	40 *	50 *	15	25	45	65	45	75
Vitamin D (mcg/d) ^b,c^	10	10	15	15	15	15	15	15
Vitamin E (mg/d) ^d^	4 *	5 *	6	7	11	15	11	15
Vitamin K (mcg/d)	2.0 *	2.5 *	30 *	55 *	60 *	75 *	60 *	75 *
Thiamin (mg/d)	0.2 *	0.3 *	0.5	0.6	0.9	1.0	0.9	1.2
Riboflavin (mg/d)	0.3 *	0.4 *	0.5	0.6	0.9	1.0	0.9	1.3
Niacin (mg/d) ^e^	2 *	4 *	6	8	12	14	12	16
Vitamin B_6_ (mg/d)	0.1 *	0.3 *	0.5	0.6	1.0	1.2	1.0	1.3
Folate (mcg/d) ^f^	65 *	80 *	150	200	300	400	300	400
Vitamin B_12_ (mcg/d)	0.4 *	0.5 *	0.9	1.2	1.8	2.4	1.8	2.4
Pantothenic Acid (mcg/d)	1.7 *	1.8 *	2 *	3 *	4 *	5 *	4 *	5 *
Biotin (mcg/d)	5 *	6 *	8 *	12 *	20 *	25 *	20 *	25 *
Choline (mg/d) ^g^	125 *	150 *	200 *	250 *	375 *	400 *	375 *	550 *
Calcium (mg/d)	200 *	260 *	700 *	1000 *	1300 *	1300 *	1300 *	1300 *
Chromium (mcg/d)	0.2 *	5.5 *	11 *	15 *	21 *	24 *	25 *	35 *
Copper (mg/d)	200 *	220 *	340 *	440 *	700	890	700	890
Fluoride (mcg/d)	0.01 *	0.5 *	0.7 *	1 *	2 *	3 *	2 *	3 *
Iodine (mcg/d)	110 *	130 *	90	90	120	150	120	150
Iron (mg/d)	0.27 *	11	7	10	8	15	8	11
Magnesium (mg/d)	30 *	75 *	80	130	240	360	240	410
Manganese (mg/d)	0.003 *	0.6 *	1.2 *	1.5 *	1.6 *	1.6 *	1.9 *	2.2 *
Molybdenum (mcg/d)	2 *	3 *	17	22	34	43	34	43
Phosphorus (mg/d)	100 *	275 *	460	500	1250	1250	1250	1250
Selenium (mcg/d)	15 *	20 *	20	30	40	55	40	55
Zinc (mg/d)	2 *	3	3	5	8	9	8	11
Linoleic Acid (g/d)	4.4 *	4.6 *	7 *	10 *	10 *	11 *	12 *	16 *
α-Linolenic Acid (g/d)	0.5 *	0.5 *	0.7 *	0.9 *	1.0 *	1.1 *	1.2 *	1.6 *

Adapted from: National Academies of Sciences, E.; Medicine. Dietary Reference Intakes for Sodium and Potassium; The National Academies Press: Washington, DC, 2019; doi:10.17226/25353 (Accessed on 15 June 2023), pp. 594. https://www.ncbi.nlm.nih.gov/books/NBK545442/ (Accessed on 15 June 2023) [125]. * The values represent recommended dietary allowances (RDAs) in bold type and adequate intakes (Ais) in ordinary type followed by an asterisk (*). ^a^ as retinol activity equivalents (RAEs). 1 RAE = 1 µg retinol; ^b^ as cholecalciferol, where 1 µ cholecalciferol = 40 IU vitamin D; ^c^ under the assumption of minimal sunlight; ^d^ as α-tocopherol; ^e^ as niacin equivalents (NE), where 1 mg of niacin = 60 mg of tryptophan; ^f^ as dietary folate equivalents (DFE), where 1 DFE = 1 µg food folate; ^g^ although AIs have been set for choline, there are few data to assess whether a dietary supply of choline is needed at all stages of the life cycle.

**Table 3:** Serum or plasma micronutrient normal laboratory values.

Micronutrient	Age	Normal	Insufficiency	Deficiency
Vitamin A (mg/L)				
	0–1 mos	0.18–0.50		<0.10
	2 mos–12 y	0.20–0.50		<0.10
	13–17 y	0.26–0.70		<0.10
Vitamin D (ng/mL)				
	All ages	30–80	20–29	<20.0
Vitamin E (mg/L)				
	0–1 mos	1.0–3.5		
	2–5 mos	2.0–6.0		
	6 mos–1 y	3.5–8.0		
	2–12 y	5.5–9.0		
	>13 y	5.5–18.0		
Vitamin K (nmol/L)				
	All ages	0.22–4.88		
Vitamin C (µmol/L)				
	All ages	23–114		
Selenium (µg/L)				
	All ages	23–190		
Zinc (µg/L)				
	All ages	60–120		
Omega 3 (mmol/L)				
	All ages	0.12–0.55		

Values obtained from Andropoulos, D.B. Appendix B: Pediatric Normal Laboratory Values. In Gregory’s Pediatric Anesthesia, 2012; https://doi.org/10.1002/9781444345186.app2 (Accessed on 15 June 2023) [148].

**Table 4:** Clinical studies that evaluated clinical outcomes of micronutrient levels and supplementation in lung disease.

Micronutrient	Lung Disease	Study Design	Age Group	Findings
Vitamin A	Pneumonia	Meta-analysis	Infants and children < 5 y (*n* = 1,202,382)	Supplementation with Vit A decreased morbidity and mortality [140].
Vitamin A	Cystic fibrosis	Observational longitudinal	Children and adolescents (*n* = 231)	Serum retinol levels were positively associated with predicted FEV1% [105].
Vitamin A	ARDS	Cross-sectional	Adults (*n* = 87)	Critically ill hospitalized patients had significantly lower Vit A levels vs. those who were moderately ill [210].
Vitamin C	Pneumonia	Meta-analysis	Children and adults (*n* = 11,306), (*n* = 2655)	Supplementation with Vit C showed a decrease in the incidence of pneumonia and decreased hospital length of stay (LOS) and mortality [128,130].
Vitamin C	Critically ill children	Prospective case-control study	Children (*n* = 81)	The prevalence of Vit C deficiency in critically ill children was 18% compared to 0% in the control group [183].
Vitamin C and Zinc	Asthma	Cross-sectional	Children aged 7–17 y (*n* = 76)	Vit C deficiency was associated with severe asthma, and plasma zinc levels were correlated with FEV1% [157].
Vitamin D	Respiratory tract infections	Observational and case-control	Infants and children (*n* = 13), (*n* = 1582), (*n* = 197), (*n* = 34), (*n* = 1016)	Insufficient or deficient plasma levels of Vit D were associated with more complications and severity of the disease [131,132,133,134,135].
Vitamin D	Pneumonia	Meta-analysis	Infants and children < 5 y. (*n* = 1601)	Supplementation with Vit D showed no difference in the duration of illness, hospital LOS, or mortality [136].
Vitamin D	Influenza	Randomized control trial (RCT)	Infants and children (*n* = 334), (*n* = 247), (*n* = 2244)	Supplementation with Vit D decreased the incidence of influenza and respiratory tract infections [137,138,139].
Vitamin D	Cystic fibrosis	Retrospective	Infants and children up to 18 y (*n* = 69), (*n* = 130), (*n* = 148), (*n* = 190)	Children with Vit D deficiency vs. those with insufficient or sufficient levels had a higher rate of pulmonary exacerbations [145,149], and serum levels of 25-OHD were lower in children colonized with *P. aeruginosa* vs. non-infected patients. [98]. Vit D supplementation with higher levels showed improved pulmonary function by predicted forced expiratory volume in 1 s (FEV1%) [146,149].
Vitamin D	Asthma	Meta-analysis	Children (*n* = 13,160)	Fifty-five percent of children with asthma were either Vit D-deficient or -insufficient, and asthmatic children were 3.4 times more likely to be Vit D-deficient vs. controls [158].
Vitamin D	Asthma	Case-control	Children aged 2–12 y (*n* = 140)	Children with moderate-to-severe asthma vs. mild asthma were 2.8 times more likely to have Vit D insufficiency [160].
Vitamin D	Asthma	Prospective, longitudinal	Children aged 7–17 y (*n* = 141)	Children with severe asthma vs. mild-to-moderate asthma were 3 times more likely to have Vit D insufficiency [161].
Vitamin D	Asthma	RCT	Children aged 6–16 y (*n* = 192), (*n* = 60)	Children supplemented with Vit D vs. placebo did not have better asthma control measured as the number of episodes, duration of the episodes, emergency visits, hospital admissions, or use of steroids [156,162].
Vitamin D	Bronchiolitis	Prospective	Children aged < 2 y (*n* = 182)	Children with low levels of Vit D had a higher degree of severity of illness and admission to the intensive care unit [169].
Vitamin D	Bronchiolitis	Case-control	Children aged 1–25 months. (*n* = 129)	Vit D status was not associated with risk of hospitalization for uncomplicated acute lower respiratory infection [170].
Vitamin D	Bronchiolitis	Retrospective	Infants (*n* = 1016)	In infants with lower levels of Vit D, presence of Hemophilus-microbiota in the nasopharynx was associated with higher severity [171].
Vitamin D	Bronchiolitis	Observational longitudinal	Children aged 3 to 6 y (*n* = 363)	Children with higher Vit D status at age 3 compared to those with lower status had decreased FEV1% and FVC% at 6 y [172].
Vitamin D and Zinc	Bronchiolitis	Double-blind, RCT	Children aged 2 to 23 months (*n* = 94)	By the third day of hospitalization, no significant difference on respiratory rate among the three groups (Control vs. Vit D vs. Zinc) and no difference in hospital LOS [173].
Vitamin D	ARDS	Systematic review, retrospective	Adults (*n* = 39), (*n* = 15,207)	Vit D deficiency was associated with increased duration of infection, ICU LOS, and mechanical ventilation [191,192].
Vitamin E	Cystic fibrosis	Observational longitudinal	Children and adolescents (*n* = 232)	FEV1% was not associated with serum α-tocopherol levels [102].
Vitamin E	Asthma	Meta-analysis	Children (*n* = 12,878)	Vit E supplementation was not associated with wheezing or asthma [159].
Omega 3	Cystic fibrosis	Meta-analysis	Children and adults (*n* = 106)	Inconclusive results with omega 3 supplementation regarding lung function or antibiotic use [154].
Selenium	Bronchiolitis	Case-control	Infants (*n* = 59)	Infants had lower levels of selenium during the hospital admission vs. post-discharge and compared to healthy controls [176].
Thiamine	Critically ill children	Prospective cohort study	Children (*n* = 202)	Low thiamine levels were found in 28% of children and was associated with high levels of *C*-reactive protein concentrations [182].
Zinc	Pneumonia	Prospective, RCT, and systematic review	Infants and children < 5 y (*n* = 320), (*n* = 103), (*n* = 100), (*n* = 5193)	Decreased zinc levels in children with higher severity of illness [141]; children who received zinc supplementation recovered faster and had shorter hospital LOS [142,143], and zinc supplementation was associated with lower incidence and prevalence of pneumonia [144].
Zinc	Cystic fibrosis	Cross-sectional	Children and adults (*n* = 53)	FEV1% was lower but not significant in patients with zinc deficiency vs. patients with normal levels [151].
Zinc	Cystic fibrosis	Double-blind placebo-controlled trial	Children aged 7–18 y (*n* = 26), (*n* = 40)	Zinc supplementation in deficient children decreased the days of oral antibiotics/year vs. the placebo group [152]; another study reported no difference [153].
Zinc	Asthma	Prospective observational	Children (*n* = 67)	Children with controlled asthma vs. the uncontrolled group had significantly higher serum zinc levels [164].
Zinc	Acute lower respiratory tract infection (ALRTI)	Meta-analysis	Children < 5 y (*n* = 1066)	There was no significant difference between placebo and zinc group regarding time to resolution of severe illness and hospital LOS [174].
Zinc	Bronchiolitis	Randomized control trial	Children aged 2–23 months (*n* = 100)	Zinc supplementation could improve clinical symptoms and decrease LOS in bronchiolitis [175].
Zinc and Selenium	Asthma	Case-control	Children aged 2–15 y (*n* = 160)	Children with asthma vs. controls had significantly lower serum levels of zinc and selenium [163].
Zinc and Selenium	ARDS	Retrospective	Adults (*n* = 22)	Zinc levels were significantly lower in severe COVID-19 induced adult respiratory distress syndrome (ARDS) [208].
Antioxidants *	Cystic fibrosis	Meta-analysis	Children and adults (*n* = 924)	Increased FEV1% in the antioxidant group vs. the control group [155].
HAT **	Septic Shock and acute lung injury	Retrospective propensity score-matched analysis	Children (*n* = 557)	Children treated with HAT therapy had lower mortality when compared with matched untreated control patients and matched hydrocortisone-only-treated patients [185].

* Included: Vit A, Vit C, Vit E, beta carotene, selenium, and glutathione; ** HAT (hydrocortisone, ascorbic acid, and thiamine).
